# Heritability of meiotic restitution and fertility restoration in haploid triticale

**DOI:** 10.1007/s00299-019-02462-6

**Published:** 2019-08-31

**Authors:** Sylwia Oleszczuk, Natalia Grzechnik, Annaliese S. Mason, Janusz Zimny

**Affiliations:** 1 Department of Plant Biotechnology and Cytogenetics, Plant Breeding and Acclimatization Institute-National Research Institute, Radzikow, 05-870 Blonie, Poland; 2 Department of Robotics and Mechatronics, AGH University of Science and Technology, al. Mickiewicza 30, 30-059 Krakow, Poland; 3 Department of Plant Breeding, Justus Liebig University, Research Center for Biosystems, Land Use and Nutrition (IFZ), Heinrich-Buff-Ring 26-32, 35392 Giessen, Germany

**Keywords:** Meiotic restitution, Unreduced gametes, Polyploidization, Doubled haploid (DH), Meiosis, Triticale

## Abstract

**Key message:**

A single division meiosis mechanism of meiotic restitution is incompletely penetrant but significantly associated with restored fertility in triticale haploids (*n* = 21, genome formula ABR).

**Abstract:**

Meiotic restitution, or failure of meiosis to produce gametes with a reduced chromosome number, can lead to the restoration of fertility in allohaploids. Meiotic restitution is of major interest for producing doubled haploids, as haploid plants undergoing meiotic restitution can often form seeds without the need to apply mitosis inhibitors to double chromosome number. We aimed to characterize meiotic restitution in a population of 183 haploids (*n* = 21, genome formula ABR) derived from an F_1_ wheat-rye hybrid where one parent was known to carry factors responsible for restoration of fertility in wide-cross haploids. Based on cytological analysis, approximately half of the plants analyzed were characterized by normal meiosis, while half showed at least some cytological evidence of meiotic restitution. However, this mechanism was incompletely penetrant in the population, with no individual plant showing 100% unreduced gamete formation: restitution occurred sectorially within each anther and was not observed in all the anthers of a given plant. Hence, the absence of meiotic restitution could not be confirmed conclusively for any individual plant, confounding this analysis. However, cytological observation of meiotic restitution was significantly associated with seed set, further confirming the role of meiotic restitution in fertility restoration. Our results provide insight into this mechanism of unreduced gamete formation, and provide a basis for future work identifying the genetic factors responsible for this trait.

## Introduction

Polyploidization (increase in the number of genomes present) is a significant evolutionary mechanism in all organisms. Increasing the number of copies of genes allows organisms to manipulate, modify, and recruit these genes into new functions. Polyploidy events have also been detected at the bases of large branches of the evolutionary tree of life (Wood et al. 2009; Jiao et al. 2011), further supporting the fundamental role of duplication of genes or genomes in speciation and diversification. However, in the short-term gene and genome, duplication usually leads to genome instability, causing changes in chromosome structure and disruption to meiosis and reproduction (Chen 2007). In autopolyploids, the presence of more than one pair of homologous chromosomes impedes chromosome segregation during meiosis, often leading to the production of unbalanced gametes and a reduction in fertility. In allopolyploid plants, similar problems can occur due to mispairing and segregation of homeologous (ancestrally related but not homologous) chromosomes (Pelé et al. 2018). Despite these problems, the addition of an extra genome or set of chromosomes has the potential to increase resistance to disease, facilitate adaptation to changing environmental conditions, and have a wide-ranging influence on agricultural phenotypes (Leitch and Leitch 2008).

The primary mechanism by which natural polyploids arise is thought to be via unreduced gametes (Harlan and DeWet 1975). In comparison to natural meiosis, as a result of which four haploid cells (reduced gametes, “n”) are obtained, unreduced gametes are formed when meiosis results in only two cells with the somatic chromosome number (“2n”). This type of meiosis was named restitution, as it returns the same number of chromosomes in the gamete as are present in the parent organism (Ramanna and Jacobsen 2003). Unreduced gamete formation is almost ubiquitous across plant and animal taxa, and is usually observed at levels of 0.1–2% of all gametes produced, with a significant influence of environmental and genetic factors on frequency and types of unreduced gamete production (Ramsey and Schemske 1998). Two main types of restitution are distinguishable by different genetic consequences: first division restitution (FDR) occurs when meiosis fails to separate homologous chromosomes, whilst second division restitution (SDR) occurs when meiosis fails to separate sister chromatids (Bretagnolle and Thompson 1995). Numerous mechanisms can result in either of these two outcomes, both with and without recombination (Bretagnolle and Thompson 1995; De Storme et al. 2013), and in practice, loss of chromosomes and other intermediate forms of meiotic restitution can also occur, leading to gametes with different chromosome complements than expected (Chester et al. 2012; Zhang et al. 2013; Oleszczuk and Lukaszewski 2014). In cereals, omission of either the first or second meiotic division is commonly observed (Silkova et al. 2011).

Unreduced gametes are more frequently produced by interspecific hybrids than natural species (Ramsey and Schemske 1998). In a typical interspecific hybrid, meiosis is seriously disrupted, because the chromosomes do not have homologues which are capable of pairing (forming bivalents). As a result of this, distribution of univalent chromosomes across the metaphase plate during meiosis I can force formation of a restitution nucleus (De Storme et al. 2013; De Storme and Mason 2014). Thus, a reduction of the chromosome number does not occur in the first meiotic division, as univalents cannot connect properly to the fibers of the karyokinetic spindle, and subsequently end up distributed across the whole cell. The effect of this division is the creation of two nuclei which usually do not contain complete genomes. The failure of univalents to align along the metaphase plate is due to the sister chromatid centromeres remaining fused in a single unit at metaphase I, only allowing attachment to one spindle pole. Meiosis in such hybrids still mostly consists of two divisions, but their products (i.e., gametes) only rarely contain a complete haploid chromosome complement, such that the hybrid is usually sterile. In cereals, another mechanism has been observed which is critical for restoring fertility by producing high frequencies of euploid unreduced gametes (Zhang et al. 2007, 2010; Silkova et al. 2013). Sometimes referred to as “single division meiosis”, the production of FDR-type gametes by alignment of univalents in the first division along the metaphase plate followed by sister chromatid separation and exit from meiosis has been observed in diverse interspecific hybrid types between *Triticum*, *Aegilops,* and *Secale* species (reviewed by Silkova et al. 2011); including *Triticum* spp. × *Aegilops* spp., *Triticum aestivum *× *Secale cereal,* and *Secale cereal *× *Aegilops squarrosa* (Sasakuma and Kihara 1981; Xu and Dong 1992; Xu and Joppa 1995). This mechanism was further observed to be dependent on all or almost all chromosomes being present as univalents, and inhibited by the presence of homologues (Wang et al. 2010; Silkova et al. 2013; Fakhri et al. 2016). Genetic factors have also been implicated, with differences between genotypes and chromosome substitution lines of species such as *Triticum turgidum * in the frequency of single division meiosis and other meiotic restitution mechanisms (Silkova et al. 2011).

In the current study, we assessed meiotic restitution and fertility characteristics in a population of microspore-derived haploids (by anther culture) obtained from an F_1_ hybrid derived from a cross between a hexaploid triticale (1605/07) and a durum wheat (Do1) × rye hybrid “MAD510”, of which “Do1MAD510” was previously observed to frequently produce self-fertile wide-cross progeny). Understanding the mechanisms of meiotic restitution and increasing its frequency of occurrence could lead to the elimination of the use of antimitotic compounds, and significant reduction of costs and effort during the production of new triticale varieties and interspecific hybrids for wheat and rye breeding, as well as shedding light on the mechanisms of polyploidisation via unreduced gamete formation in interspecific hybrids.

## Materials and methods

### Plant growth and material

The “Do1” line, formed by crossing *T. turgidum* ssp. *persicum* (2n = 4*x* = AABB = 28) with *T. turgidum* ssp. *dicoccoides* (2n = 4*x* = AABB = 28), was selected in IPG PAS, Poznan by Dr. B. Łapiński for its capacity to produce self-fertile F_1_ hybrids with rye. Neither of its parent genotypes exhibited the ability to produce unreduced gametes during meiosis by themselves. However, later observations showed self-fertility manifesting itself in all tested wide hybrids derived from Do1 as a parent. F_1_ hybrids were generated by intercrossing the Do1 line of tetraploid wheat (*Triticum turgidum* L.) with diploid rye (*Secale cereale* L.; 2n = 2*x* = RR = 14) variety ‘MAD510’. The ‘MAD510’ population is a winter rye homozygous for an introgression of a segment of wheat chromosome 1D on the long arm of rye chromosome 1R (Lukaszewski et al. 2000). Otherwise, it was created by crossing individuals from three varieties of rye: “Motto”, “Amilo”, and “Dańkowskie Złote”.

Pollen from hybrid F_1_Do1MAD510 was used to pollinate hexaploid triticale line “1605/07” (2n = 6*x *= AABBRR); this variety is derived from “Presto” and carries the FC2 chromosome (Lukaszewski 2006). Hybrid F_1_ plants (putatively 2n = 6*x* = AABBRR) from the combination 1605/07 × Do1MAD510 were used as the starting material to obtain haploid regenerants (*n* = 21 = 3*x *= ABR) by in vitro androgenesis in anther cultures.

Donor plants and androgenic regenerants were grown in the greenhouse at the IHAR-PIB in Radzikow, Poland, and as well as at the campus of the University of California, Riverside, California, USA.

### In vitro androgenesis and regeneration

After microscopic analysis to establish that microspores were at late developmental stages, the tillers of the greenhouse-grown donor plants were collected. To maintain high humidity, the experimental material was wrapped in cellulose foil and stored in Erlenmeyer flasks with tap water in dark conditions at 4 °C for 3 weeks. Following removal of tillers from the spikes, anthers were sterilized and then placed on petri dishes with solidified induction medium 190-2 (Zhuang and Xu 1983) supplemented with 90 g/l maltose, 438 mg/l glutamine, 2 mg/l 2.4-D, and 0.5 mg/l kinetin, and cultured in the dark at 26 °C. Individual androgenic structures (calli, embryos, and embryo-like structures) were manually selected at different times, transferred onto regeneration medium 190-2 supplemented with growth regulators according to  Pauk et al. (1991), and cultured under 16/8 h (day/night) photoperiod. The procedure was repeated several times after 1–2 weeks as new androgenic structures appeared. After reaching about 2 cm, obtained green plants were relocated into Erlenmeyer flasks with medium according to Pauk et al. (1991) and later, upon developing roots, potted. Following their adaptation to soil, they were vernalized for 6 weeks at 4 °C, after which they were grown in a greenhouse under standard conditions until maturity. All emerging heads of each individual were bagged to prevent uncontrolled pollination. Plant fertility was evaluated on the basis of developed seed set.

### Ploidy-level analysis

Measurements of ploidy levels were taken from the population of androgenic plants. Flow cytometry analysis was conducted on samples prepared according to Galbraith et al. (1983) with some modifications. Young leaf fragments from regenerants were chopped into pieces with a razor blade in 2 ml of the nuclei isolation buffer [0.1 M Tris, 2.5 mM MgCl_2_ × 6H_2_O, 85 mM NaCl, 0.1% (v/v) Triton X-100] containing DAPI (1 µg/ml). The obtained cell suspension was filtered through a 30 nm mesh nylon filter. Samples of nuclei were tested using a Partec CAII flow cytometer. Ploidy levels were determined by comparing G1 peaks of the analyzed samples with internal control samples (leaf tissue of the hexaploid triticale). Aneuploid plants were not considered.

### FDR presence evaluation

#### Cytological analysis

Spikes of regenerants potentially going through meiosis (the sheath of the flag leaf above the lowest leaf and the tip of the spike reaching the level of the lowest leaf) were harvested and dissected. One anther from each spikelet was freshly squashed in a drop of 2% acetocarmine and analyzed under a microscope. If the desired stages of meiosis were present, the remaining two anthers of the spikelet were fixed in a mixture of three parts absolute alcohol to one part glacial acetic acid for 1 week at 37 °C and stored frozen at − 18 °C until needed. During the initial as well as the later analysis, all meiocytes on the slides were assessed.

#### Phenotypic analysis

To identify any relationships between the presence/absence of meiotic restitution and later stages of plant development, a subset of the regenerant population (183 plants) was studied at anthesis and maturity. During anthesis, the presence and absence of bursting anthers and pollen grain release was observed, and at the later stage of maturity, whether or not seeds were formed.

### Statistical analysis

Associations between self-pollinated seed production, anther bursting and observed meiotic restitution mechanisms were assessed using Pearson’s *χ*^2^ contingency tables on expected vs. observed numbers in each category (fertile vs. non-fertile, restitution-observed vs. no-restitution-observed). Significant deviation from expected ratios of fertile vs. non-fertile and restitution-observed vs. no-restitution-observed plants was also assessed using Pearson’s *χ*^2^ test (*p* < 0.05).

## Results

### Identification and intercrossing of partners with differing production of FDR-type gametes

Hybrid “F_1_Do1MAD510” (2n = ABR, derived from the cross AABB “Do1” × RR “MAD510”) was found to be self-fertile and able to produce FDR-type gametes. Another hexaploid triticale line “1605/07” (2n = 6*x *= AABBRR) did not demonstrate the ability to produce FDR-type gametes as an allohexaploid (2n = AABBRR). These two lines (ABR hybrid “F_1_Do1MAD510” and triticale “1605/07”) were subsequently intercrossed to produce F_1_-hybrids that were then used for androgenesis via anther culture.

### Rates of androgenesis and plant regeneration in anther culture of triticale hybrids

**Fig. 1:**
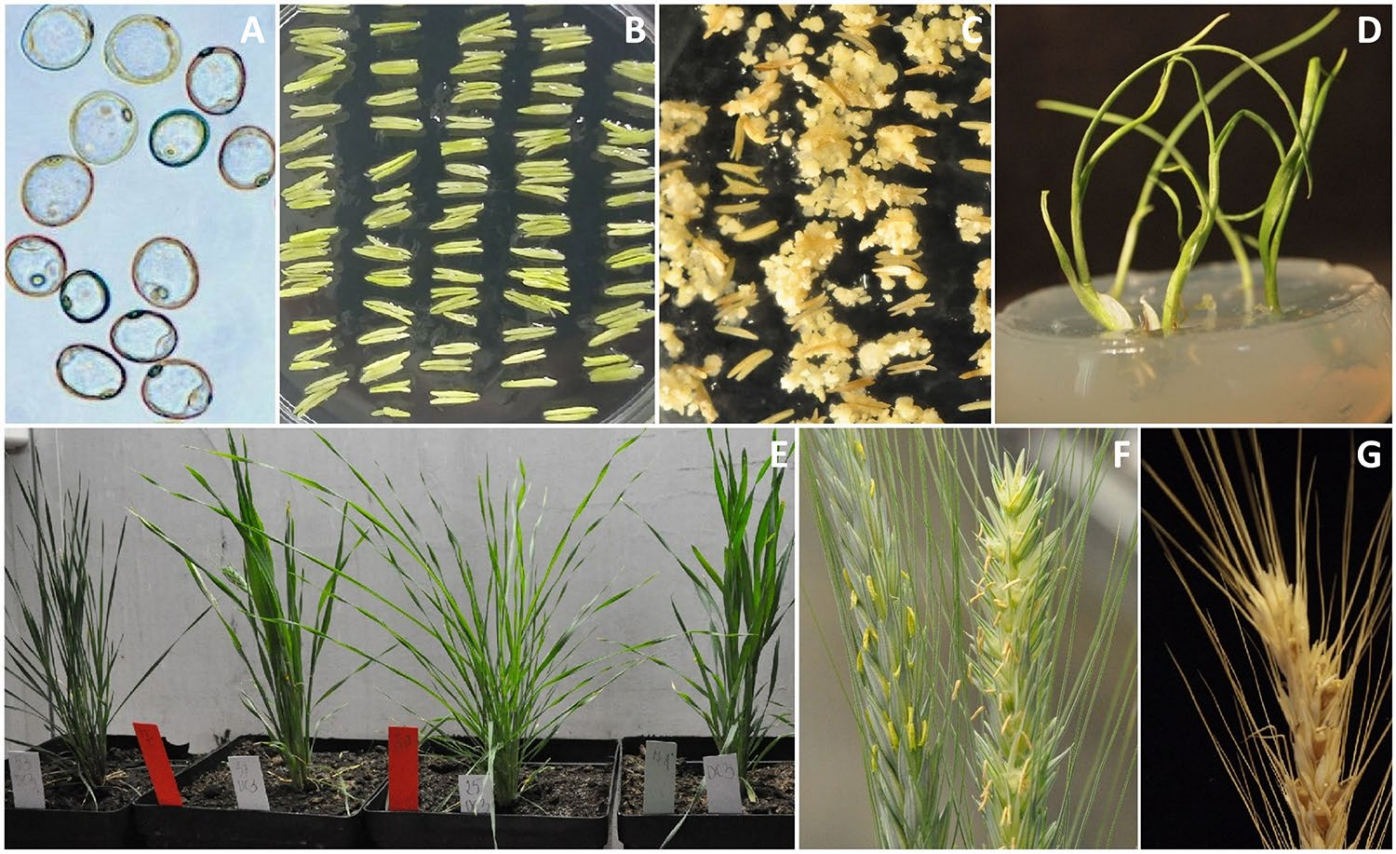
Primary stages in haploid plant regeneration via anther culture: **a** late-stage microspores, scale bar 20 μm; **b** freshly isolated anthers on the induction medium, scale bar 5 mm; **c** embryos and calli, scale bar 5 mm; **d** regenerated plantlets on rooting medium, scale bar 1 cm; **e** differentiation in tillering-stage regenerants, scale bar 10 cm; **f** flowering in haploids, scale bar 4 cm; and **g** a fertile microspore-derived haploid, scale bar 2 cm

The androgenesis process was carried out twice during two subsequent seasons. The parameters of the process were only monitored in the first experiment, as the main purpose of the project was to generate populations of doubled haploid (DH) triticale lines for genetic mapping via androgenesis in anther culture. For the purposes of this manuscript, androgenic regenerants which were triploids (three genomes—ABR, 21 chromosomes) due to the nature of their haploid-like behavior (lack of partners for homologous chromosome pairing) have been referred to as haploids. On average, 140 anthers were isolated from each of the 85 spikes used. The efficiency of androgenesis induction as measured by the number of reacting anthers reached 70%. Despite the large number of androgenic structures and the high rate of their conversion into plants, the efficiency of green plant regeneration per spike was 4.1 on average (maximum 22 green plants produced per spike). The reason for such low efficiency was the large percentage of albino regenerants. In the first experiment, 351 green regenerants were obtained, of which spontaneous DHs accounted for 21% (73 plants). Among the post-phenotypic analysis regenerants, up to 36% (128 plants) of the plants were aneuploids and about 3% did not reach maturity. In the second experiment, 113 plants were produced, of which 22% (25 plants) were DH and the rest were haploids (88 plants). Only plants with the gametic (haploid; *n* = 21 = 3*x* = ABR) chromosome number were used for cytological studies of FDR-like restitution mechanisms. As a result, a total of 183 individuals from both experiments which were confirmed to be haploid by flow cytometry and cytology were singled out for detailed cytological analysis. Primary stages in haploid plant regeneration via anther culture are presented in Fig. 1.

### Cytological analysis-using live-staining and fixed material reveals FDR is highly sectorial

Androgenic regenerants were subjected to cytological analysis to assess the frequency of the FDR-like restitution processes. Both fresh and fixed anthers were analyzed. Live-stained material allowed the observation of entire columns of meiocytes and thus not only the presence or absence of restitution, but also their relative proportions. In addition, live-stained material allowed the analysis of the presence and absence of cell sectors undergoing various disturbances or stages of meiosis (as meiosis within each anther was not synchronized). However, it was difficult to observe individual cells in the meiocyte columns or to photographically document this unstained material. Fixed material slides allowed more detailed analysis of scattered meiocytes from the entire anther, but gave little information on the frequency of FDR. In fixed material it was also impossible to assess whether the presence of certain meiocyte stages (i.e., dyads) was the result of restitution or normal meiosis. However, the combination of both approaches gave satisfactory results.

During the early stages of testing, it became apparent that FDR is a sectorial phenomenon, meaning that the affected meiocytes only occurred in some anthers and rarely along the entire length of the column. Occasionally, only a single cell in the entire anther was undergoing restitution, while the rest of the meiocytes were observed to be normal for a hybrid meiosis. A similar tendency was noticed during bursting of anthers and seed formation. However, the release of pollen grains also did not necessarily lead to seed formation, possibly as a result of failure of the female gametophyte to produce FDR-derived gametes, which was impossible to observe prior to seed formation.

### Two types of meiosis were observed in the ABR plants

**Fig. 2:**
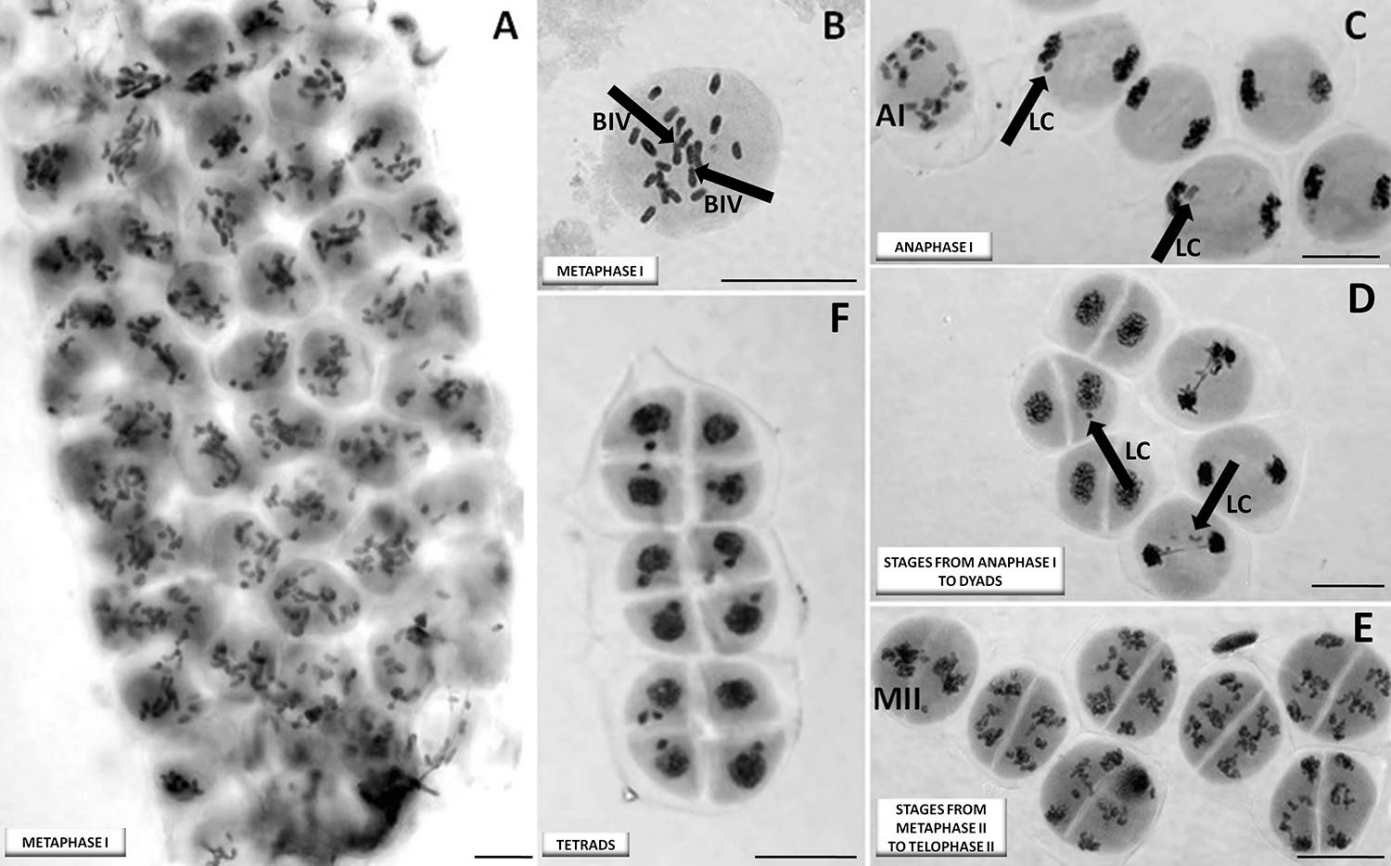
“Normal” meiosis in a microspore-derived haploid (*n* = 21, ABR) from a triticale hybrid: **a** column containing meiocytes in metaphase (live-stained material); **b** a single meiocyte after fixation, with two bivalents (BIV) (marked with arrows) and univalents scattered across the metaphase plate; **c** meiocytes in anaphase I (labeled as AI), one cell in earlier anaphase I and five in later anaphase I: laggard univalents (LC) are marked with arrows, and two chromatids are visible in a laggard univalent at the bottom of the image; **d** stages from anaphase I to dyads: laggard univalents (LC) are marked with arrows; **e** stages of metaphase II (labeled MII) and telophase II; noticeable differences in the resulting cell nucleus sizes and two dyads are observed due to unequal separation of univalents in anaphase I, with many laggard chromosomes visible on the metaphase plate; **f** the tetrad stage, where noticeable differences in the cell nucleus size are observed, and 10 out of 12 daughter cells contain micronuclei resulting from laggard univalent in anaphase II. Scale bar 40 μm

Two clearly different meiotic processes were observed in the ABR plants. In “normal” interspecific hybrid meiosis, metaphase I could only be differentiated from anaphase I in meiocytes when at least one (homeologous) bivalent was formed, as most univalents remained unpaired during meiosis in the ABR plants. Bivalents formed in this manner were observed to attach to the metaphase plate, surrounded by disorganized univalents (Fig. 2a, b). Anaphase II could be identified by the presence of separating sister chromatids which were still connected by their centromeres. As the separation of univalents in the first division was irregular, resulting nuclei (e.g., dyads and tetrads) clearly differed in size. There were frequent cases of delayed separation of chromosomes where laggard chromosomes remained on the metaphase plate in metaphase II and subsequently formed micronuclei (Fig. 2c, d). Tetrads were usually formed as a result of the second meiotic division (Fig. 2e). Among 183 analyzed plants, 64 showed only “normal” meiosis of this type (Table 1).

**Table 1:** Cytological observations of meiosis and seed set in 183 analyzed haploid triticale plants (*n* = 21, ABR)

FDR^a^ observed	Seed formation	Number of plants	Total number of seeds	Average number of seeds per plant	Number of seeds in the plant
+	+	51	1248	24.5	from 1 to 155
+	−	42	0	0	0
−	+	26	432	16.6	from 1 to 192
−	−	64	0	0	0

^a^First Division Restitution meiosis

**Fig. 3:**
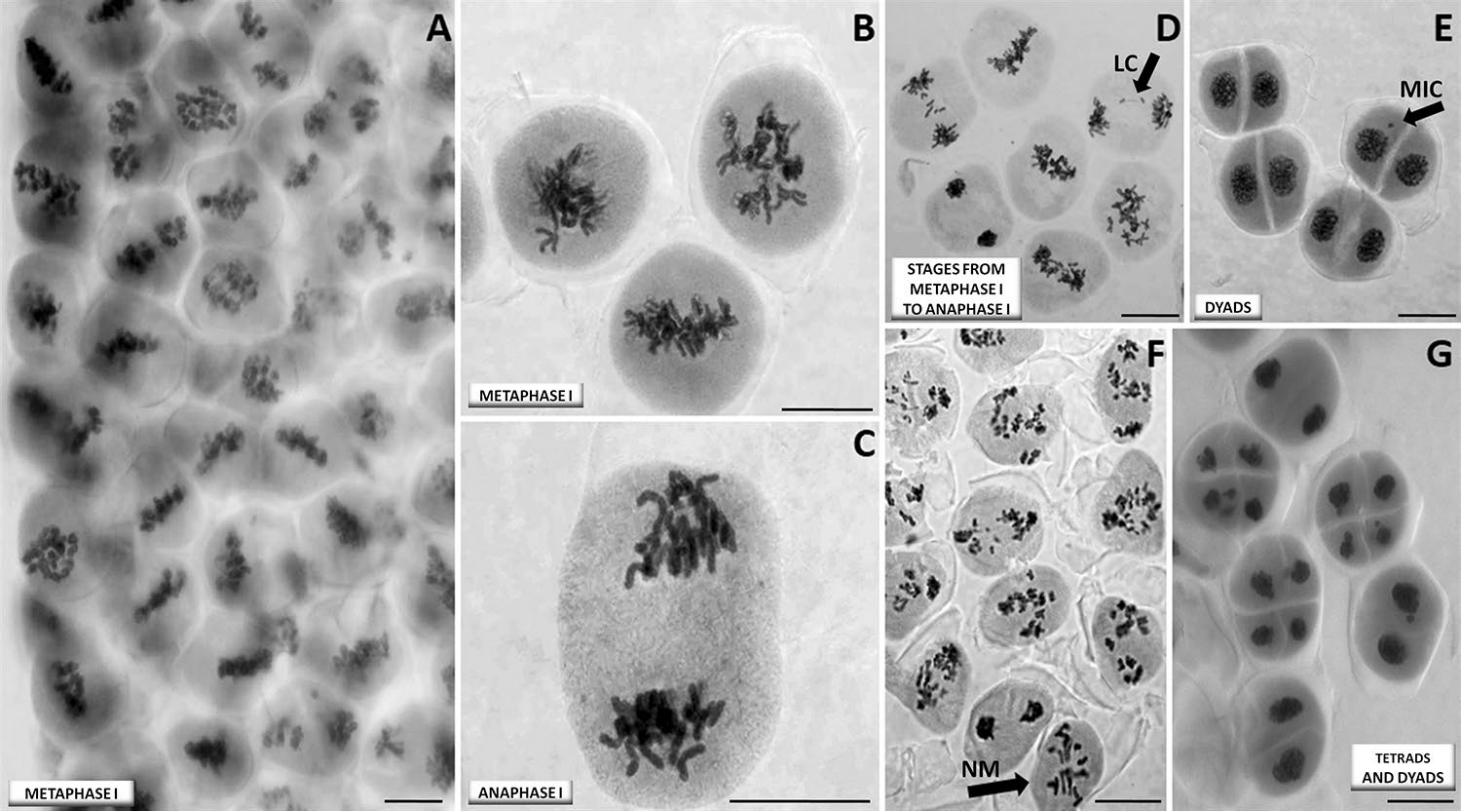
First division restitution (FDR) of meiosis in a microspore-derived haploid (*n* = 21, ABR) from a triticale hybrid: **a** column of meiocytes with typical signs of FDR, i.e., univalents positioned on the metaphase I plate, and separating sister chromatids in anaphase (live-stained material); **b** a close-up view of meiocytes in metaphase I with an equatorial and a polar view of the metaphase plate; **c** a meiocyte in anaphase I, where all chromosomes at the pole clearly comprise sister chromatids; **d** stages from metaphase I (four meiocytes) to various stages of anaphase I, showing clear sister chromatid separation and the presence of laggard chromosomes (LC) (marked with an arrow); **e** dyads at the final stage of division, where similar nuclei sizes are observed in each dyad, along with a rare micronucleus (MIC) (marked with an arrow) putatively resulting from laggard chromosomes; **f** the sectorial nature of FDR—a portion of the meiocyte column is shown where the meiocyte marked with an arrow is undergoing “normal” meiotic division (NM), while the remaining meiocytes show division with restitution; and **g** the final products of meiotic division: three tetrads and three dyads. Scale bar 40 μm

In “abnormal” interspecific hybrid meiosis, FDR-type gametes were produced after omission of normal metaphase I. In the cells undergoing FDR, most univalent bipolar were attached to the spindle to form the metaphase plate in the first (only) division, which made the FDR process easy to identify (Fig. 3a–e). Among the 183 cytologically analyzed plants, not a single one had all of its meiocytes undergoing FDR. As mentioned previously, initiation of the FDR process was observed to be sectorial, ranging from single meiocytes to specific sections of the meiocyte columns (Fig. 3f, g).

**Fig. 4:**
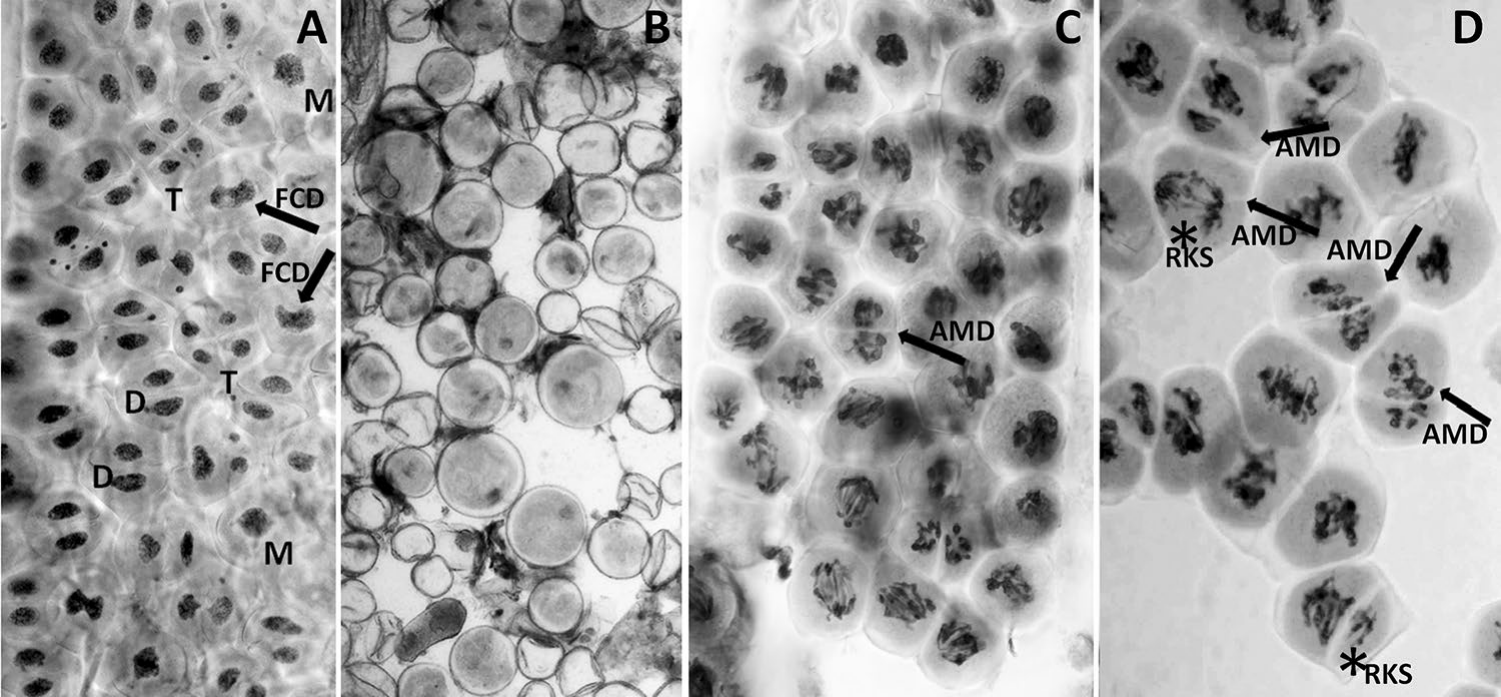
Undefined type of meiotic division in a microspore-derived haploid (*n* = 21, ABR) from a triticale hybrid: **a** the column of meiocytes containing the final products of meiotic division: a mixture of monads (marked with M), dyads (marked with D), tetrads (marked with T), and a few cell nuclei that did not undergo meiotic division (FCD) (marked with arrows), scale bar 40 μm; **b** noticeable differences in pollen grain size, scale bar 20 μm; **c** and **d** the column of meiocytes in stages similar to prophase I and anaphase I, where meiocytes seem to form a residual karyokinetic spindle (RKS) (marked with an asterisk) which makes normal separation of sister chromatids difficult, and potentially impossible. This type of meiosis exhibits many features found in FDR. It is possible that this division is extremely delayed, or cytokinesis is accelerated; however, in many cases, there are signs of attempted cell division in an unusual plane, often across the accumulated chromosomes on the metaphase plate, or at unusual angles. The arrows mark an example of this unusual type of division (AMD). Scale bar 40 μm

The typical signs of single division meiotic restitution were observed in 93/183 plants: univalents were arranged on the metaphase plate at right angles to the long axis of the spindle, often with already-separated sister chromatids still connected by centromeres. The proportions of meiocytes with restitution were not estimated. Monads and dyads were also present at the tetrad stage. In a few cases, some of the univalents migrated prematurely to the poles, while others remained on the metaphase plate preparatory to separation of the sister chromatids. Giant pollen grains were also often observed in the mature pollen fraction, and most likely contained double the number of chromosomes (Fig. 4a, b).

Anomalies were observed in the division of seven plants, including a process of “misdivision division” similar to that described by Shamina et al. (1999). In these plants, the meiocytes seemed to have been unable to form the karyokinetic spindle. Some of these meiocytes showed no sign of spindle presence (although no attempt was made to specifically dye the spindle), and these meiocytes did not divide and thus formed monads. Some of the other unusual cases included only partial spindle formation, leading to incomplete division of the nucleus, and the development of a thick, multi-chromosomal chromatid bridges towards the end of meiosis (Fig. 4c).

### Associations between FDR, anther bursting, and seed formation

**Fig. 5:**
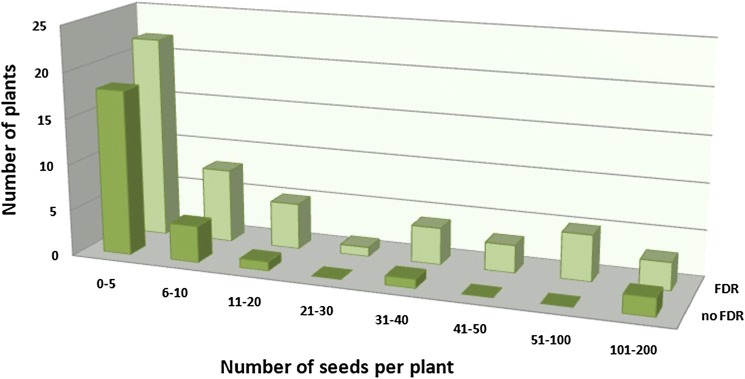
Results from cytological observation (first division restitution; FDR) and seed formation among 183 analyzed haploid triticale plants

Plant classification with regards to restitution was only possible after direct, cytological observation. The analysis was performed on 183 plants, among which FDR was found in 93 (Fig. 5). In addition, seed formation was observed in 26 specimens, but the process of the restitution was not found cytologically (Table 1). However, due to the sectorial nature of the phenomenon, the lack of visible cells undergoing restitution did not have to mean its absence. In those cases, anther bursting and seed formation may also indicate the presence of the disorder. However, there was no strict interdependence between the cytologically observed restitution, anther bursting, and seed formation. Moreover, 30 of the plants with observed restitution did not produce seeds, and 14 out of 44 cytologically confirmed restitution-containing regenerants produced at least one kernel. Two-thirds (66%, 51/93) of plants for which FDR was observed produced seeds, while only 29% (26/90) of plants where no FDR was observed produced seeds (Table 1). Plants for which FDR was observed were significantly more likely to produce seed than plants for which FDR was not observed (Pearson’s *χ*^2^ test, *p* = 0.00038; Table 1, Fig. 2a, b). The number of plants for which FDR was observed was not significantly different from that expected from a 1:1 segregation ratio in the population (Table 1, Pearson’s *χ*^2^ test, *p* = 0.82). The number of plants for which one or both of seed formation and cytological FDR was observed did not fit a 1:1 or 3:1 segregation ratio (Table 1, Pearson’s *χ*^2^ test, *p* < 0.0001 and *p* = 0.0018, respectively), but did fit a 2:1 ratio (Table 1, Pearson’s *χ*^2^ test, *p* = 0.64).

**Fig. 6:**
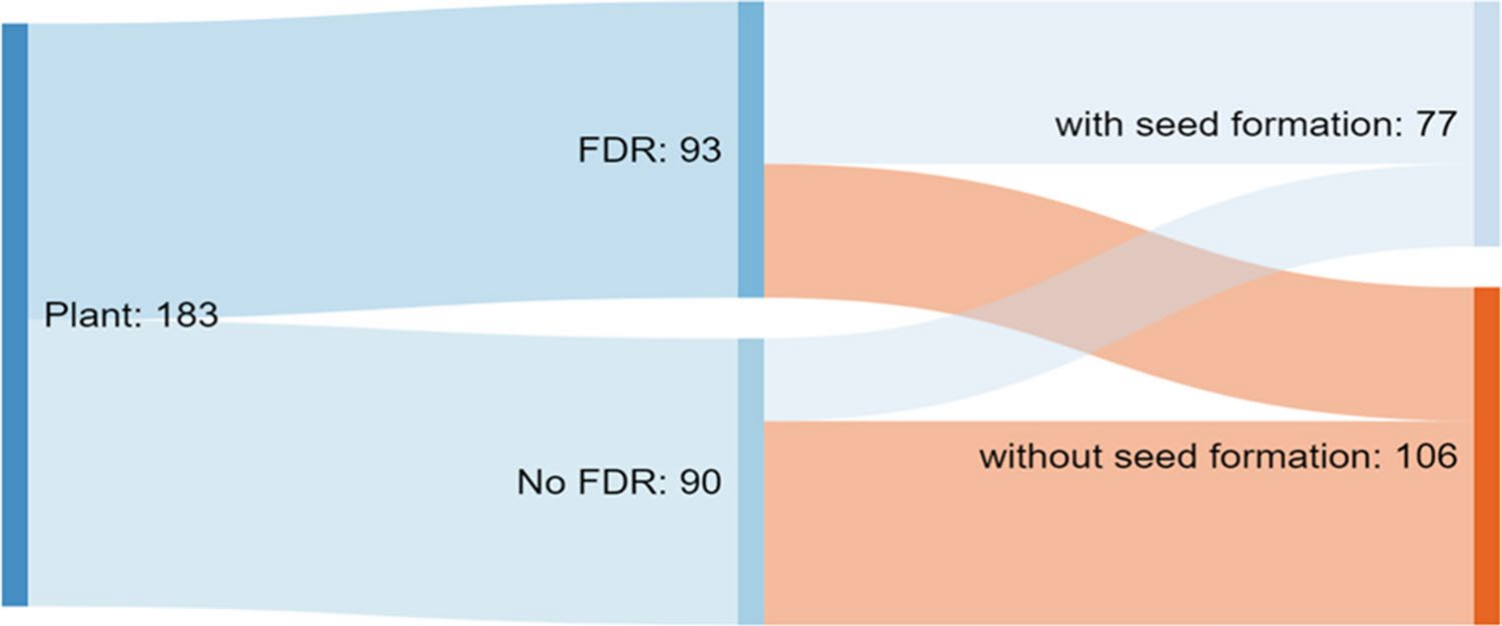
Frequency of particular seed sets in the population of haploid triticale plants with (FDR) and without (no FDR) cytologically observed first division restitution

**Fig. 7:**
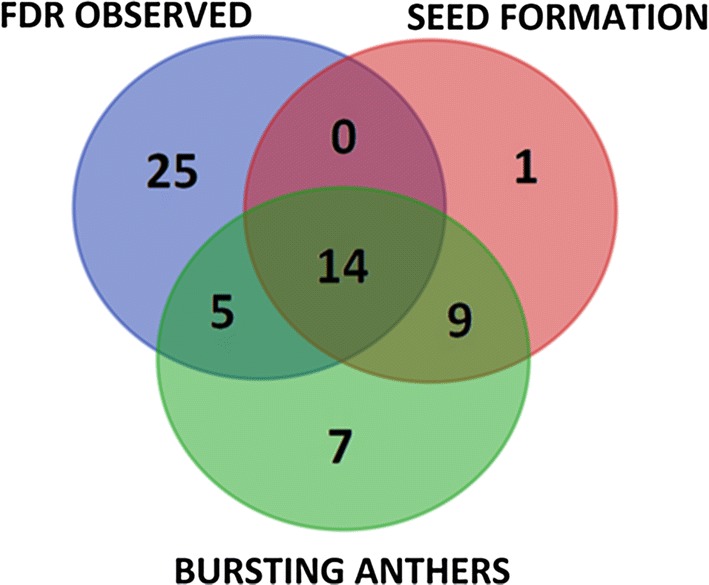
Cytological observations of first division restitution meiosis (FDR) and fertility phenotypes in 84 haploid triticale plants (*n* = 21, ABR)

Observation of anther bursting (maturation) was carried out on 84 plants, of which 35 were found to have at least one bursting anther (Fig. 6). Cytologically confirmed FDR was not significantly associated with anther bursting (Pearson’s *χ*^2^ test, *p* = 0.76), but anther bursting and seed set were significantly associated (Pearson’s *χ*^2^ test, *p* < 0.0001). Only 4% of individuals (1/24) produced seed without observation of anther bursting, while 66% of individuals (23/35) for which anther bursting was observed produced seeds. Out of all the analyzed specimens, 49 were characterized by a lack of both viable pollen and seed set (Fig. 7). The number of plants for which FDR was observed was not significantly different from expected 1:1 segregation ratio in the population (Fig. 7, Pearson’s *χ*^2^ test, *p* = 0.66), while the number of plants with any of observed FDR, bursting anthers, or seed formation fits both a 3:1 and a 2:1 ratio (Fig. 7, *p* = 0.61 and *p* = 0.25, respectively, Pearson’s *χ*^2^ test).

## Discussion

Unreduced gamete formation by interspecific hybrids is a mechanism which potentially plays a crucial role in evolution, but about which very little is known (Mason and Pires 2015). In addition, identifying factors enabling fertility in androgenic progenies could allow for exploitation of these factors for breeding or research purposes in DH lines production. In the current study, we characterized meiosis and fertility in an androgenic triticale haploid (*n* = 21 = 3*x* = ABR) population. This population was found to be segregating for production of unreduced gametes and self-pollinated seed as a result of an omission of the first division restitution meiotic mechanism. This mechanism was incompletely penetrant in the population, with no individual plant showing 100% unreduced gamete formation. Cytological observation of this mechanism was significantly associated with seed set, supporting the previously observed role of this mechanism in fertility restoration in other interspecific hybrid types (Silkova et al. 2011). A 1:1 segregation ratio in the population of a meiotic restitution-promoting factor was best supported by the data, suggesting that a single genetic locus may be responsible for this trait. However, difficulties in quantifying cytological data prevent strong conclusions being made, and this conclusion would be in contrast to previous studies identifying this restitution type as a putatively multi-locus trait (Silkova et al. 2011).

The meiotic stage when the cell decides to follow the route of restitution is not fully known. In typical meiosis, centromeres of sister chromatids are fused in a single unit, which eliminates the possibility of their interaction with opposite poles of the spindle. In certain conditions, in univalents, centromere separation could occur, such that univalents are able to divide into the sister chromatids in the first meiotic division. It is obvious that for univalents to separate sister chromatids in an organized way in FDR, their centromeres must be arranged in a different way or reorganized during the division. Until now, it has not been possible to capture the moment of such a change in the organization of centromeres. This reorganization might not occur until a stage close to metaphase: univalents in normal meiosis, which stay on the metaphase plate for a long time, separate sister chromatids with high frequency (Lukaszewski 2010), whereas univalents which arrive at the poles earlier never exhibit such behavior. As far as that in normal meiosis, it is possible to approximately define how long the metaphase lasts, i.e., how long the univalent stays on the metaphase plate, in meiosis without chromosome pairing, such an evaluation is not possible.

Cytological classification of the androgenic plants obtained was hindered by their tendency to behave as univalents in meiosis, but we observed that in a typical restitution of this type, all univalents arrange themselves on the metaphase plate in a “normal” configuration, i.e., perpendicular to the metaphase plate. However, as described by Oleszczuk and Lukaszewski (2014), even in restitution, it occurs that some of the univalents migrate prematurely to the poles without separation of sister chromatids. On the other hand, univalents in normal meiosis have a tendency to stay on the metaphase plate and then often separate the sister chromatids (Lukaszewski 2010). Meiocytes were hence difficult to classify as either “restitutional” or “non-restitutional”, since quantitative variation was observed in the number of univalents which prematurely migrated to the poles and the number of univalent which stayed on the metaphase plate and underwent sister chromatid separation.

For several reasons, the cytological analysis which was carried out did not fully characterize the tested regenerants regarding their ability to restitute. First, it was not possible to confirm the presence of the process either in all the anthers of a given plant or even more so in all the spikes of a given individual. In some plants, where the process of restitution was confirmed cytologically, and rupturing anthers were present, seed set was still not observed. This lack of correlation could be explained by the sectorial nature of the observed restitution, which could be present in only a certain part of the anther, or in just a few segments of particular anthers, leading to the creation of too small an amount of functional pollen for the anther walls to break. Apart from this, the meiotic restitution process did not necessarily occur simultaneously in the ovule mother cells in the same floret, but for the haploid to create a seed, restitution must take place in both the male and female gametes. In the case of successful seed formation, both female and male gametophytes are expected to contain meiotic restitution-derived meiocytes. However, we did observe seed production in some individuals where no-restitution or anther bursting was observed. This is most likely due to the sectorial nature of the phenomenon, and we could easily have simply missed observing these events, particularly as some anthers were left on each plant to produce seeds and later, unobserved meiosis could have contributed to seed set. Alternatively, some other forms of restitution, additional somatic doubling or other meiotic abnormalities like endoreduplication, c-mitosis, or formation of other unusual chromosome constitutions as well as some forms of apomixis could also be taken into consideration as leading to fertility (Seguí-Simarro and Nuez 2008; Chester et al. 2012; León-Martínez and Vielle-Calzada 2019). The one plant which we observed with seeds but no anther bursting could be the result of uncontrolled and unobserved cross-pollination, although we did our best to control for this, but could also simply be due to the reasons described above. Due to the significant difficulties in cytological analysis of restitution in female cells very little work has been published to date on this, although female unreduced gametes have been observed to result in seed production in other species (Ramanna and Jacobsen 2003).

The next point which deserves attention is the observation of a mix of tetrad and dyads on some microscope slides. Here, a few situations could be taking place: either some of the cells underwent normal meiosis resulting in tetrads, while in some of the cells, the second division was delayed or the cells did not enter the second division at all (in various sectors of the anther, meiosis could be at a somewhat different stage, which is a natural phenomenon) or, the mix of dyads and tetrads is a result of the fact that the dyads resulted from single division meiotic restitution. After restitution, such cells are characterized by symmetricity, i.e., the nucleus is equally divided, as a result of which the daughter cells contain the same amount of genetic material and have an identical shape. By contrast, in typical hybrid meiosis, the tetrads have a very irregular shape due to the random segregation of univalents into daughter nuclei. The shape of the resulting cells could, in this case, be a clue as to which situation is occurring, i.e., whether the division was normal but irregular, or whether it was disrupted by the FDR mechanism. However, yet again, cytological categorization in this population was unclear due to the indeterminate phenotype, making it difficult to draw conclusions.

In the research encompassed in this work, the presence of micronuclei was confirmed in most monads, dyads, and tetrads. Micronuclei have previously been observed during unreduced gamete formation in *Triticum aestivum *× *Aegilops* spp. cross hybrids (Fakhri et al. 2016). These micronuclei were probably formed from “laggard” chromosomes, in other words, those which stayed too long on the metaphase plate, and, as a result of which, were not pulled to the poles during anaphase I or anaphase II. In comparison with the research of the aforementioned authors, the presence of triads was not observed, although monads were found. The presence of monads suggests a lack of meiotic division, while the formation of triads is usually attributable to a second division-restitution mechanism by which meiosis I proceeds normally, but meiosis II fails to separate sister chromatids in one of the two nuclei groups (Bretagnolle and Thompson 1995).

The level of seed setting in haploid plants can be used as a measure of functional unreduced gamete production (Hao et al. 2014). In our study, the meiotic restitution phenomenon in haploid plants was also confirmed by the ability of these plants to form seeds. In each example, plant fertility was low, with only one to a few dozen seeds produced. The greatest probability of the occurrence of restitution was predicted from fixed anthers from plants which had formed several dozen or more seeds. In such preparations (slides), large groups of cells (approx. 60–150 cells) undergoing restitution were observed more often, as opposed to the majority of preparations where only isolated individual cells undergoing restitution were observed. Regenerants which had not formed seeds were also found despite the clear observation of meiotic restitution in those cells, but only for preparations where meiotic restitution was restricted to isolated cells. Therefore, the number of cells in which meiotic restitution is present could have an influence on whether a plant forms seeds, such that seed setting requires the presence of a greater number of cells in which restitution occurs. Apart from this, taking into account the large proportion of aneuploids among the tested individuals, it is also possible to assume that sterility of the hybrid does not mean a lack of restitution. In drawing conclusions from our results, it is necessary to consider that hybrid meiosis is irregular and differs from typical meiosis, and as such irregularities resulting from the hybrid meiosis may appear similar to restitution. For example, dyad production due to segregation of univalent chromosomes to a single pole during meiosis could be typical low-frequency outcome for abnormal meiosis without the single division restitution mechanism in the hybrid, and even occasionally result in seed production, as previously observed in ABDR hybrid types (Zhang et al. 2007).

Genotype-specific effects on seed formation via self-pollination in durum wheat by *A. tauschii* hybrids have been observed previously (Xu and Joppa 2000; Zhang et al. 2008), suggesting that unreduced gamete formation is under genetic control. As a result of our research, a mapping population was created which will enable the localization of loci responsible for the process of meiotic restitution. If the effects of particular genetic regions on the trait of meiotic restitution via omission of the first meiotic division can be recognized and confirmed, and the genes responsible for restitution correctly identified, it will be possible to consider the practical exploitation of this phenomenon in the doubling of chromosome numbers in haploids. DHs in triticale and in many other crops are of widespread use in breeding in creating homozygous lines to facilitate genetic selection, but most commonly, it is necessary to use colchicine to obtain them. Skillful exploitation of the natural mechanism of restitution could improve the whole breeding process. Our results also provide rare data on the putative evolutionary mechanisms of unreduced gamete formation, lending weight to the hybrid bridge hypothesis for allopolyploid speciation (Ramsey and Schemske 1998).

## References

[CR1] Bretagnolle F, Thompson JD (1995). Gametes with the somatic chromosome number: mechanisms of their formation and role in the evolution of autopolyploid plants. New Phytol.

[CR2] Chen ZJ (2007). Genetic and epigenetic mechanisms for gene expression and phenotypic variation in plant polyploids. Annu Rev Plant Biol.

[CR3] Chester M, Gallagher JP, Symonds VV (2012). Extensive chromosomal variation in a recently formed natural allopolyploid species, *Tragopogon miscellus* (Asteraceae). Proc Natl Acad Sci.

[CR4] De Storme N, Mason A (2014). Plant speciation through chromosome instability and ploidy change: cellular mechanisms, molecular factors and evolutionary relevance. Curr Plant Biol.

[CR5] De Storme N, De Schrijver J, Van Criekinge W (2013). GLUCAN SYNTHASE-LIKE8 and STEROL METHYLTRANSFERASE2 are required for ploidy consistency of the sexual reproduction system in Arabidopsis. Plant Cell.

[CR6] Fakhri Z, Mirzaghaderi G, Ahmadian S, Mason AS (2016). Unreduced gamete formation in wheat × *Aegilops* spp. hybrids is genotype specific and prevented by shared homologous subgenomes. Plant Cell Rep.

[CR7] Galbraith DW, Harkins KR, Maddox JM (1983). Rapid flow cytometric analysis of the cell cycle in intact plant tissues. Science.

[CR8] Hao M, Luo J, Zeng D, et al (2014) QTug.sau-3B is a major quantitative trait locus for wheat hexaploidization. G3 Genes, Genomes, Genetics 4(10):1943–534. 10.1534/g3.114.013078 10.1534/g3.114.013078 PMC4199700 25128436

[CR9] Harlan J, DeWet J (1975). On Ö. Winge and a prayer: the origins of polyploidy. Bot Rev.

[CR10] Jiao Y, Wickett NJ, Ayyampalayam S (2011). Ancestral polyploidy in seed plants and angiosperms. Nature.

[CR11] Leitch AR, Leitch IJ (2008). Genomic plasticity and the diversity of polyploid plants. Science.

[CR12] León-Martínez G, Vielle-Calzada J-P (2019). Apomixis in flowering plants: developmental and evolutionary considerations. Curr Top Dev Biol.

[CR13] Lukaszewski AJ (2006). Cytogenetically engineered rye chromosomes 1R to improve bread-making quality of hexaploid triticale. Crop Sci.

[CR14] Lukaszewski AJ (2010). Behavior of centromeres in univalents and centric misdivision in wheat. Cytogenet Genome Res.

[CR15] Lukaszewski AJ, Brzezinski W, Klockiewicz-Kaminska E (2000). Transfer of the Glu-D1 locus encoding high molecular weight glutenin subunits 5 + 10 from breadwheat to diploid rye. Euphytica.

[CR16] Mason AS, Pires JC (2015). Unreduced gametes: meiotic mishap or evolutionary mechanism?. Trends Genet.

[CR17] Oleszczuk S, Lukaszewski AJ (2014). The origin of unusual chromosome constitutions among newly formed allopolyploids. Am J Bot.

[CR18] Pauk J, Manninen O, Mattila I (1991). Androgenesis in hexaploid spring wheat F2 populations and their parents using a multiple-step regeneration system. Plant Breed.

[CR19] Pelé A, Rousseau-Gueutin M, Chèvre A-M (2018). Speciation success of polyploid plants closely relates to the regulation of meiotic recombination. Front Plant Sci.

[CR20] Ramanna MS, Jacobsen E (2003). Relevance of sexual polyploidization for crop improvement—a review. Euphytica.

[CR21] Ramsey J, Schemske DW (1998). Pathways, mechanisms, and rates of polyploid formation in flowering plants. Annu Rev Ecol Syst.

[CR22] Sasakuma T, Kihara H (1981). A synthesized common wheat obtained from a triploid hybrid, *Aegilops squarrosa* var. strangulata × *Triticum durum*. Wheat Inf Serv.

[CR23] Seguí-Simarro JM, Nuez F (2008). Pathways to doubled haploidy: chromosome doubling during androgenesis. Cytogenet Genome Res.

[CR24] Shamina N, Dorogova N, Goncharov N (1999). Abnormalities of spindle and cytokine behavior leading to the formation of meiotic restitution nuclei in intergeneric cereal hybrids. Cell Biol Int.

[CR25] Silkova OG, Shchapova AI, Shumny VK (2011). Meiotic restitution in amphihaploids in the tribe Triticeae. Russ J Genet.

[CR26] Silkova OG, Adonina IG, Krivosheina EA (2013). Chromosome pairing in meiosis of partially fertile wheat/rye hybrids. Plant Reprod.

[CR27] Wang CJ, Zhang LQ, Dai SF (2010). Formation of unreduced gametes is impeded by homologous chromosome pairing in tetraploid *Triticum turgidum* × *Aegilops tauschii* hybrids. Euphytica.

[CR28] Wood T, Takebayashi N, Barker M (2009). The frequency of polyploid speciation in vascular pants. Proc Natl Acad Sci USA.

[CR29] Xu S, Dong Y (1992). Fertility and meiotic mechanisms of hybrids between chromosome autoduplication tetraploid wheats and Aegilops species. Genome.

[CR30] Xu SJ, Joppa LR (1995). Mechanisms and inheritance of first division restitution in hybrids of wheat, rye, and *Aegilops squarrosa*. Genome.

[CR31] Xu SJ, Joppa LR (2000). First division restitution in hybrids of Langdon durum disomic substitution lines with rye and *Aegilops squarrosa*. Plant Breed..

[CR32] Zhang LQ, Yen Y, Zheng YL, Liu DC (2007). Meiotic restriction in emmer wheat is controlled by one or more nuclear genes that continue to function in derived lines. Sex Plant Reprod.

[CR33] Zhang L, Chen Q, Yuan Z, Xiang Z, Zheng Y, Liu D (2008). Production of aneuhaploid and euhaploid sporocytes by meiotic restitution in fertile hybrids of durum wheat Langdon lines with *Aegilops tauschii*. J Genet Genomics.

[CR34] Zhang LQ, Liu DC, Zheng YL (2010). Frequent occurrence of unreduced gametes in *Triticum turgidum*-*Aegilops tauschii* hybrids. Euphytica.

[CR35] Zhang H, Bian Y, Gou X (2013). Persistent whole-chromosome aneuploidy is generally associated with nascent allohexaploid wheat. Proc Natl Acad Sci.

[CR36] Zhuang JJ, Xu J, Hu H, Vega MR (1983). Increasing differentiation frequencies in wheat pollen callus. Cell and tissue culture techniques for cereal crop improvement.

